# Overexpression of PLXDC2 in Stromal Cell-Associated M2 Macrophages Is Related to EMT and the Progression of Gastric Cancer

**DOI:** 10.3389/fcell.2021.673295

**Published:** 2021-05-28

**Authors:** Yiming Guan, Yuzhang Du, Guanzheng Wang, Hongquan Gou, Yilun Xue, Jingsong Xu, Enhao Li, David W. Chan, Di Wu, Peiqing Xu, Peihua Ni, Dakang Xu, Yiqun Hu

**Affiliations:** ^1^Faculty of Medical Laboratory Science, Ruijin Hospital, School of Medicine, Shanghai Jiao Tong University, Shanghai, China; ^2^Department of Obstetrics and Gynaecology, LKS Faculty of Medicine, The University of Hong Kong, Hong Kong, China

**Keywords:** tumor-associated M2 macrophages, gastric cancer, plexin domain containing 2 (PLXDC2), stroma, EMT

## Abstract

The tumor microenvironment (TME) comprises distinct cell types, including stromal types such as fibroblast cells and macrophage cells, which have recently become a critical factor in tumor development and progression. Here, we identified the TME-related gene, plexin domain containing 2 (PLXDC2), in a high-stromal-score population. And we revealed that this gene was related to poor survival and advanced (tumor-node-metastasis) stage in gastric cancer (GC) patients from The Cancer Genome Atlas database. An integrated gene profile and functional analysis of the proportions of tumor-infiltrating immune cells revealed that the expression of the M2 macrophages cell marker CD163 was positively correlated with PLXDC2 expression. In addition, the M2 macrophages gene signature and high PLXDC2 expression were associated with the inflammatory signaling pathway and the epithelial-to-mesenchymal transition (EMT)-related gene signature. Single-cell study of GC identified PLXDC2 was enriched specifically in fibroblasts and monocytes/macrophages populations, which supported its important role in the stroma. Furthermore, according to a tissue microarray immunohistochemistry analysis, the expression of PLXDC2 elevated in human GC stromal specimens compared to tumor tissue specimens. Moreover, PLXDC2 overexpression in the stromal compartment was associated with CD163-positive regulatory M2 macrophages, and its functions were related to the pathogenesis of GC. Multiplexed immunohistochemistry verified PLXDC2’s correlation with EMT markers. Our data suggested that PLXDC2 was expressed in stromal cells and that its crosstalk with tumor-associated macrophages could contribute to cancer biology by inducing the EMT process.

## Introduction

Gastric cancer (GC) is one of the major causes of cancer-related mortality worldwide. It has been reported that 1.0337 million new GC cases and approximately 783,000 GC- related deaths occurred in 2018 (Bray et al., 2018; Siegel et al., 2021). This may be attributed to the high *Helicobacter pylori* (HP) infection rate (Hatakeyama, 2019; Kotilea et al., 2019). In total, around 679,000 new GC cases and 498,000 GC-related deaths occur each year in China; these values are second only to those of lung cancer, indicating that GC is a major health issue (Chen et al., 2016; Zong et al., 2016). Although recent studies have proposed new treatment strategies based on disease risk stratification [tumor-node-metastasis (TNM) stage] and histological grade according to tumor size, lymph node or distant metastasis (also AJCC system, *T* describes the size of the original tumor and whether it has invaded nearby tissue, *N* describes nearby lymph nodes that are involved, and *M* describes distant metastasis; Edge and Compton, 2010), the ability of these strategies to improve the prognosis of GC patients has been widely questioned. Further studies on the diagnosis and treatment of this type of malignancy are required. Molecular diagnosis has been previously proven to be effective. Discoveries of biomarkers, such as ERBB2 and EGFR, aid in informed treatment decision making by medical oncologists (Yasui et al., 2001). Innovations related to molecular biomarkers are likely to improve the prognosis and treatment response of GC patients. Thus, a study seeking novel GC molecular biomarkers might offer a solution to our current challenges.

Alteration of the tumor microenvironment (TME) has been proven to be a pivotal strategy for improving GC prognosis (Wu and Dai, 2017). Studies have shown that TME influences the therapeutic response and clinical outcome. A recent comprehensive analysis of the TME of GC, comprising 1524 GC samples, depicted the landscape of GC’s TME characteristics. The results proved that the TME of GC patients could help predict the response to immunotherapies and provide new strategies for treating GC (Zeng et al., 2019). The non-tumor components of TME, including borders, extracellular matrix (ECM), stromal cells, blood vessels, lymph vessels, and immune/inflammatory cells, have been recognized as affecting GC patients’ prognosis. By dynamically interacting with tumor cells, stromal cells participate in all stages of tumorigenesis, progression, metastasis, recurrence and drug response, thereby affecting the fate of patients. In the process of tumor evolution and metastasis, the stromal cells in TME have also undergone some changes and played a role in the inhibition and promotion of metastasis, and the overall function of stromal cells is conducive to survival and migration of cancer cells. Immune-related cells also play a decisive role in cell fate. Tumor suppressive immune cells, such as CD8^+^ T cells, and tumor progressive immune cells, such as regulatory T cells, are directly connected to tumor growth. Among all types of immune cells of different functions, T cells and macrophages, are most abundant in the TME. T cells’ mechanisms of cytotoxicity have been explored in numerous studies, whereas the behavior patterns of macrophages are less well understood. Therefore, a macrophages- related TME study of GC has the potential to provide novel insight into this malignancy.

Macrophages, which are believed to be an essential type of immune cell during inflammation, can both promote and suppress tumor growth (Lewis and Pollard, 2006; Szebeni et al., 2017). M1 macrophages have been labeled as antitumor factors, whereas M2 macrophages have been identified as protumor factors (Yunna et al., 2020). Persuasive evidence at both clinical and experimental levels indicates that M2 macrophages can promote cancer initiation and malignant progression (Jackaman et al., 2017). Nevertheless, the complexity of immune cell behaviors led us to ambiguity in macrophages M2-related characteristics. Although previous studies have explored the immune-related functions of M2 macrophages, few explorations have tried to determine whether M2 macrophages can affect tumor growth by other crucial mechanisms, including Epithelial-to-mesenchymal transition (EMT) or angiogenesis. Clarifying this particular immune cell type’s behaviors might aid in improving the prognosis of tumors such as GC.

Epithelial-to-mesenchymal transition has been proven to be a survival-related program (Zhang and Weinberg, 2018). Its function of increasing the motility or invasiveness of cancer cells suggests its involvement in metastasis, which contributes to high mortality rates among cancer patients (Pastushenko and Blanpain, 2019). EMT is a cell biological program that transforms epithelial cells to more mesenchymal cell-like cells. In a sense, the occurrence of EMT indicates the malignant process of tumors. Decreased expression of E-cadherin and cytokeratin and increased expression of N-cadherin, Twist and Zeb1 is a common EMT program signature (Krebs et al., 2017). Studies on EMT are required to better understand cancer metastasis and to gain control over this process that increases malignant potential.

Plexin domain containing 2 (PLXDC2), also known as TEM7R, is a member of the tumor endothelial marker (TEM) family, which is associated with tumor-specific angiogenesis. PLXDC2 has been identified as a regulator of several brain activities, including the coordinated control of proliferation and cell fate specification along and across the neuroaxis (Miller-Delaney et al., 2011). Studies of PLXDC2 and its closest homolog, PLXDC1, have been conducted in cancers, including breast cancer, colorectal cancer, and typical liver cancer types. They are recognized as possible targets of treatment and have certain prognostic value (Nanda et al., 2004; Rmali et al., 2005; Yamamoto et al., 2020). However, none of the previous studies examined the linkage between PLXDC2 and GC. While PLXDC2 demonstrates a clear linkage with stroma-related behaviors, the relationship between PLXDC2 and the EMT program remains unknown; however, clarification of this relationship could provide a potential breakthrough in terms of GC prognosis.

In the current study, we utilized the accessible GC patient data from The Cancer Genome Atlas (TCGA) stomach adenocarcinoma (STAD) dataset alongside an additional Gene Expression Omnibus (GEO) GSE84437 GC dataset. The ESTIMATE algorithm was used to explore stromal and immune characteristics, and PLXDC2, a gene not previously studied in the context of GC, was identified to be related to the disease. Further studies on this gene were conducted. Gene Set Enrichment Analysis (GSEA) and the novel tool CIBERSORTx provided clear evidence that PLXDC2 affects the EMT program and inflammatory response and is linked to protumor M2 macrophages. An integration of Single-cell analysis and IHC study provided solid evidence that PLXDC2 upregulation in stroma-related cells contribute to poor survival. The utilization of multiplexed immunohistochemistry (mIHC) verified PLXDC2’s correlation with EMT markers. Thus, the PLXDC2/EMT/M2 macrophages axis was established.

## Materials and Methods

### Data Acquisition

RNA-seq data of 375 GC samples and 32 matched non-cancerous samples were downloaded from TCGA. Clinical data, including age, sex, tumor grade, clinical stage, TNM stage, and survival time, were also downloaded. Data extraction was conducted with R software 4.0.3 (R Foundation for Statistical Computing, Vienna, Austria). A subsidiary GC tissue dataset was downloaded from the GEO. The GSE84437 dataset (Yoon et al., 2020), containing Illumina HumanHT-12 V3.0 beadchip microarray expression series matrix files based on the GPL6947 platform, was selected. The Single-Cell RNA samples used in this study were from a previous single cell analysis study of GC^1^ (Sathe et al., 2020).

### Analysis of the Immune Score and Stromal Score of the TME

ESTIMATE^2^ (Yoshihara et al., 2013) is a well-established algorithm that generates immune and stromal scores based on RNA-seq data. A high score indicates a large proportion of the corresponding component in the TME. In this research, the ESTIMATE algorithm was employed via the “estimate” package 1.0.13 of R software.

### Analysis of the Correlation Between Survival and Immune/Stromal Score

A total of 371 TCGA tumor samples and 433 GEO tumor samples were adequate for survival analysis. Kaplan–Meier plots of patients from datasets grouped by both stromal score and immune score were generated via GraphPad Prism 8.0 software with log-rank *p* < 0.05 as the indicator of statistical significance.

### Identifying Differentially Expressed Genes Between High- and Low-Immune/Stromal Score Groups

Samples from the TCGA and GEO datasets were sorted into high score groups and low score groups according to the median immune score and stromal score. “limma” package 3.44.3 (Ritchie et al., 2015) of the Bioconductor project (Huber et al., 2015) was then utilized to determine the differences in gene expression between the high score group and the low score group. Genes with a | log2(fold change)| > 1 and a false discovery rate (FDR) < 0.05 were considered significantly differentially expressed and were selected for subsequent analysis. Heatmaps of differentially expressed genes (DEGs) were then generated via the “pheatmap” package 1.0.12.

### Analysis of the Correlation of Stromal Score With Clinical Stage

The correlation of the stromal score with the clinicopathological characteristics in the TCGA dataset was explored via GraphPad Prism software. One-way ANOVA (Analysis of Variance) was conducted, and statistical significance was indicated by a *p* value less than 0.05.

### Cox Regression Analysis

The “survival” package 3.2-7 of R software was used for univariate Cox regression analysis of the TCGA samples. Genes that passed the univariate Cox test are shown in the plot.

### Exploring DEGs and Their Correlations With Survival

The second version of Gene Expression Profiling Interactive Analysis (GEPIA2)^3^ (Tang et al., 2019) was utilized to determine the differences in expression between tumor samples and normal gastric samples from the TCGA and Genotype-Tissue Expression (GTEx) databases. A Kaplan–Meier survival plot was also generated via Kaplan–Meier Plotter^4^ (Szasz et al., 2016) using GC gene chip data. A receiver operating characteristic (ROC) curve was generated via the “survival ROC” R package 1.0.3.

### Gene Set Enrichment Analysis

Gene Set Enrichment Analysis was performed to investigate whether there were significant differences in the expression of the identified genes between the two groups. Hallmark gene sets v7.2 were downloaded as the target sets, and GSEA (Subramanian et al., 2005) was performed using GSEA_4.0.3 software. The nominal (NOM) *p* value, FDR and normalized enrichment score (NES) were used to identify the pathways enriched in each phenotype. Gene sets with NOM *p* < 0.05 and FDR *q* < 0.06 were considered significant.

### Identifying Immune Cell Profiles

The novel CIBERSORTx^5^ (Steen et al., 2020) computational method was applied to estimate all tumor samples’ tumor-infiltrating immune cells (TICs) abundance profiles. In total, 289 samples passed the significance test. Survival and correlation analysis were conducted via TIMER 2.0^6^.

### Single-Cell Analysis

The Single-Cell RNA Sequencing (scRNA-seq) samples in this study were downloaded from https://dna-discovery.stanford.edu/research/datasets/. We used filtered output of Cellranger from scRNA-seq of GC, which consists of 8 patients’ cells from GC, paired normal tissue, and PBMCs (Sathe et al., 2020). All samples were input in Seurat V3, and a total of (43147/56167) cells were obtained after a restrict quality control (number of detected gene > 200, log10 Gene per nUMI > 0.8, percentage of mitochondrial genes < 20%; Stuart et al., 2019). We removed potential doublets cells by DoubletFinder in default, and assuming doublet formation rate was based on Single Cell 3’ Reagent Kits v2 User Guide^7^ (McGinnis et al., 2019). After “NormalizeData,” different samples were merged by “IntegrateData” workflow and IKAP were used to scale (batch, nUMI and percentage of mitochondrial genes), to determine nPCs (PC18K11) and to visualize in UMAP (Chen et al., 2019). NK cells and B cells were next distinguished from first determined immune cells. “FindAllMarkers” function was set (min.pct = 0.25, logfc.threshold = 1, and tes.use = “wilcox”) to find markers of all cell types. Dot plot were plotted by “Dotplot” function, and Violin plot were plotted by “Vlnplot” function.

### Pathology Analysis

Tissue microarrays (TMAs) containing 90 human GC and adjacent non-tumorous specimens were obtained from the Ruijin Hospital, School of Medicine, Shanghai Jiao Tong University from April 2008 to October 2009. All the protocols using human specimens were approved by the Ethics Committee of Shanghai Jiao Tong University. The slide was deparaffinized, rehydrated and microwaved-heated. Primary antibody, CD163, was incubated at RT for 30 min. Nuclei were stained with hematoxylin, cytoplasm with eosin. A pathologist identified the interface of tumor and stromal tissue. Image recognition for tissue-component segmentation of tumor cells and stroma regions based on spectral channel was conducted with inForm (Akoya Biosciences, Marlborough, United States). The expression levels of target proteins in tissue were examined by a pathologist blinded to the patients’ clinical characteristics. According to the fluorescence intensity threshold, the positive degree was divided into 0 (negative), 1, 2, and 3 (threshold criteria: 0.0625, 0.1250, 0.1875, and 0.2500). Images were acquired with a Vectra microscope.

### Multiplexed Immunohistochemistry and Immunofluorescence Analysis

Paraffin sections were dewaxed with xylene and gradient ethanol solution. Endogenous peroxidase blocking solution (Beyotime, Shanghai, China) was used for peroxidase blocking, and the blocking/antibody (Panovue, Beijing, China) was used for unrelated antibody blocking. The primary antibodies included anti-PLXDC2 (12285-1-AP, Proteintech, Chicago, United States), anti-E-Cadherin (ab76011, Abcam, Cambridge, United Kingdom), and anti-Vimentin (ab92547, Abcam, Cambridge, United Kingdom). Opal 4-color manual IHC kit (Akoya biosciences, MA, United States) was used for mIHC. The Vectra Polaris (PerkinElmer, Spokane, WA, United States) was used to capture the staining image. The inform software (Akoya Biosciences, Marlborough, United States) was used for data analyzing. PLXDC2 High Expression Region was defined as the region which had at least 95 percent of PLXDC2 positive cells. PLXDC2 Low Expression Region was defined as the region which has at most 5 percent of PLXDC2 positive cells. The percentage of PLXDC2, E-Cadherin and Vimentin positive cells was calculated as the number of positive cells divided by the total number of DAPI-positive cells. To quantify the expression levels, ten different fields were selected for the calculation.

### Statistical Analyses

All statistical analyses were performed in R software (version 4.0.3) and GraphPad Prism 8 software. The threshold for statistical significance for all statistical tests was *p* < 0.05. The log-rank test was utilized to assess significance for Kaplan–Meier survival analysis.

## Results

### Identification of Key Regulated Genes in GC Through the ESTIMATE Algorithm

In hopes of discovering novel regulators in GC, data including 407 samples from the TCGA STAD dataset and 433 samples from the GSE84437 dataset were downloaded and organized into two files. The ESTIMATE algorithm was conducted, and independent stromal scores and immune scores were generated. Common DEGs from the high/low immune score and stromal score comparisons were then analyzed with univariate Cox regression. Survival-related genes that were expressed differently in tumor and stromal samples were then discovered. We focused on PLXDC2, a gene that was not previously linked with GC. GSEA and CIBERSORTx analysis were conducted sequentially and proved that PLXDC2 was associated with immunity and the EMT process. Our results were ultimately confirmed with Single-Cell analysis and TMA study. The analytical procedure for the study is shown in Supplementary Figure 1.

### An Elevated Stromal Score Is Significantly Correlated With Advanced Clinicopathological Indicators and a Poor Prognosis

**FIGURE 1:**
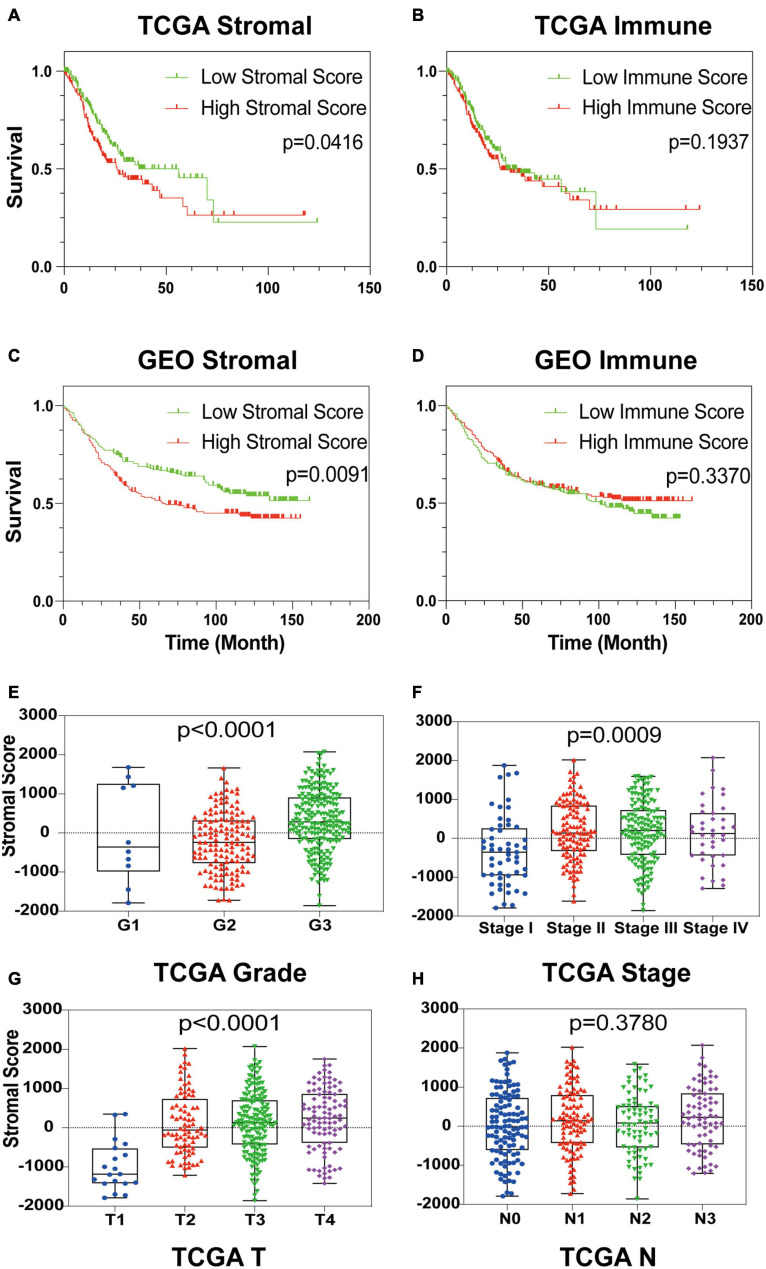
Identification of GC Stromal Scores’ linkage with prognosis and clinicopathology. **(A–D)** Kaplan–Meier survival analysis of TCGA STAD patients and GEO GSE84437 patients grouped by immune score and stromal score. The red line indicates the high ESTIMATE score group, and the green line indicates the low ESTIMATE score group. **(E–H)** The relationship between the TCGA ESTIMATE stromal score and tumor grade, clinical stage, *T* stage and *N* stage. Each dot signifies the stromal score in a sample.

To establish the linkage between estimated scores and the overall survival rate, Kaplan–Meier plots were generated. Higher respective scores indicated larger amounts of the immune or stromal components in the TME. Both datasets’ plots suggested a favorable prognosis for patients with low stromal scores, as shown in Figures 1A–D, which implied that GC stromal score is an independent prognostic indicator. A further linkage between stromal scores and the clinicopathological characteristics of GC patients was then explored. As the GEO dataset GSE84437 included limited clinicopathological data, analyses were only conducted with TCGA STAD samples. As shown in Figures 1E–H, the GC stromal score was positively correlated with several GC classifications, including tumor grade (*p* < 0.0001), clinical stage (*p* = 0.0009), and T classification of the TNM stage (*p* < 0.0001), which implied that stromal scores are associated with GC progression. The results above suggested that a high stromal score is correlated with advanced clinicopathological indicators and therefore is an adverse prognostic factor.

### Identification of PLXDC2 as a Key Regulator That Promotes GC Progression

**FIGURE 2:**
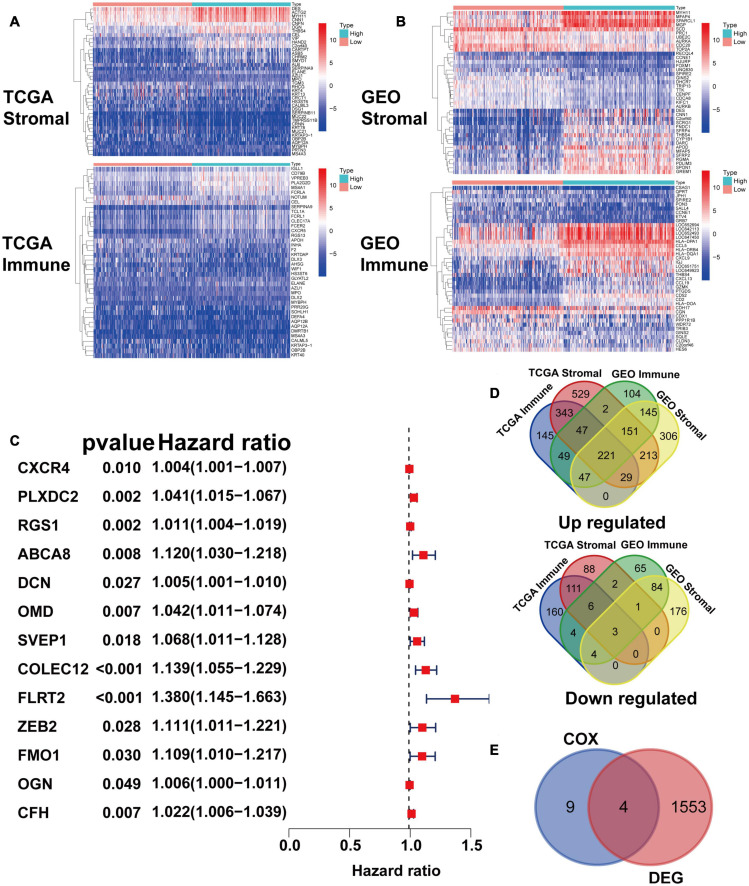
Identification of prognostic gene markers according to patients’ stromal and immune scores. **(A)** Heatmaps of the top 20 DEGs generated by comparing the high stromal/immune score group and the low stromal/immune score groups of TCGA STAD patients. The heatmaps’ row name is the gene name, and the ID of the samples is the column name, which is not shown in the plot. DEGs were determined by the Wilcoxon rank sum test with *q* = 0.05 and | fold change| (FC) > 1 after log_2_ transformation as the thresholds for significance. **(B)** Heatmaps of DEGs in GEO patients, similar to **(A)**. **(C)** Venn plots generated via an online tool (http://bioinformatics.psb.ugent.be/webtools/Venn/) depicting the up-regulated and down-regulated DEGs shared by the TCGA STAD and GEO datasets. **(D)** Forest plot of Cox regression (with 95% confidence interval), which was conducted with the 224 DEGs. **(E)** Venn plot depicting genes that were both related to survival and differentially expressed between tumor samples and normal samples.

**FIGURE 3:**
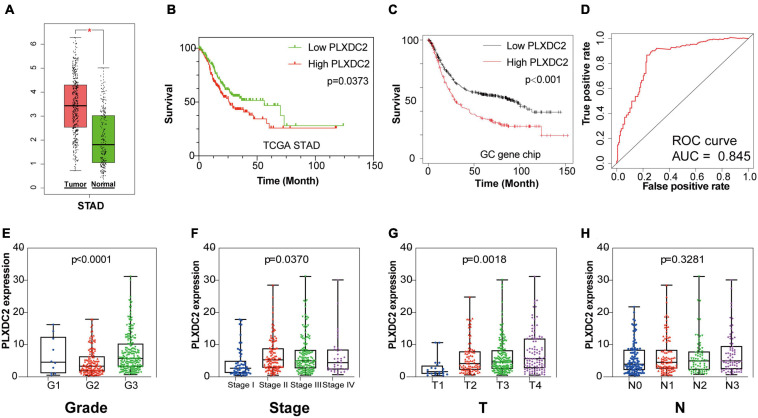
PLXDC2 is a DEG capable of propelling GC progress. **(A)** Differential expression of PLXDC2 in normal and tumor samples generated by GEPIA2. Analyses were performed across normal and tumor samples of TCGA STAD alongside GTEx normal samples. **(B)** Survival analysis of TCGA STAD patients with different PLXDC2 expression levels. Patients were sorted into high expression or low expression groups according to the median expression level (*p* = 0.0373 by log-rank test). **(C)** Survival curves of GC patients generated by Kaplan–Meier Plotter (*p* < 0.0001 by log-rank test). **(D)** ROC curve for PLXDC2. An AUC close to 1 suggests high reliability. **(E–H)** The relationship between PLXDC2 expression and tumor grade, clinical stage, *T* stage and *N* stage. Each dot signifies the PLXDC2 expression level in a sample.

After identifying the prognostic value of the stromal scores, key genes of TME associated with survival were selected. A comparison analysis between the high score group and low score group of both datasets was first carried out. A total of 224 DEGs were screened out, including 221 up-regulated and 3 down-regulated genes. Heatmaps and Venn plots were generated to demonstrate the process (Figures 2A,B,D). The selected 224 genes were then assessed by univariate Cox regression analysis, which identified 13 genes related to survival (Figure 2C). The genes finally overlapped with genes that exhibited expression differences in STAD tumor samples and normal samples. The genes with an absolute log2(fold change) value threshold of 1.6, calculated with the limma plot, were selected. This method identified four genes, namely, CXCR4, PLXDC2, ABCA8, and RGS1, identified by this method (Figure 2E). Although ABCA8 is a DEG with survival correlation, its connection was not coordinative with survival rates, as it suggests higher expression in cancer than normal while indicating better prognosis, the gene was eliminated. The three genes screened above were then examined via different methods. Given that previous studies have recognized CXCR4 and RGS1 as prognostic markers of GC, we focused our research on PLXDC2, a gene that was not previously discovered as a GC marker. Differential expression of PLXDC2 in tumor and normal samples were first demonstrated in Figure 3A, using the GEPIA2 webtool, which combined the TCGA STAD samples with the GTEx normal samples. The Kaplan–Meier survival plot is presented in Figure 3B (*p* = 0.0373). An additional Kaplan–Meier plot of patient survival based on PLXDC2 expression was then generated via the Kaplan–Meier Plotter webtool (see text footnote 4) using the mRNA gene chip data of GC (*p* < 0.0001), and the results supported the prognostic value of PLXDC2 (Figure 3C). A ROC curve for PLXDC2 was obtained (Figure 3D). The area under the curve (AUC) represents the set of all possible statistical tests of the microarray data with equal probability for a true positive and a false positive result based on each decision threshold value. The plot suggested PLXDC2 as a preferrable gene marker for indicating GC prognosis. Further correlations between PLXDC2 expression and the clinicopathological characteristics of GC patients are shown in Figures 3E–H, indicating that PLXDC2 was positively correlated with several GC classifications, namely, tumor grade (*p* < 0.0001), clinical stage (*p* = 0.0370), and T classification of TNM stages (*p* = 0.0018). These results provide solid evidence that PLXDC2 acts as a key regulator and stimulates GC progression.

### PLXDC2 Expression Was Correlated With EMT and Inflammatory Signaling Pathways

**FIGURE 4:**
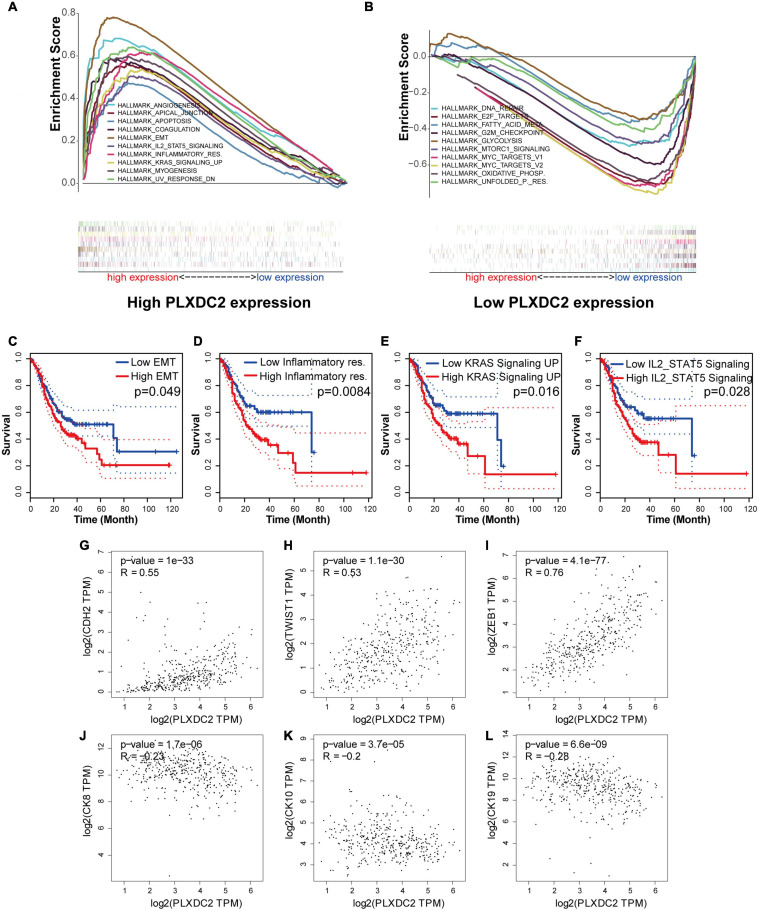
PLXDC2 is relevant to EMT and several immune-related pathways. **(A)** Up-regulated HALLMARK pathways in samples with high PLXDC2 expression. Particular gene sets are represented with lines of unique color, and up-regulated genes are located on the left approaching the origin of the coordinates, while the down-regulated genes are on the right of the *x*-axis. Only gene sets with NOM *p* < 0.05 and FDR *q* < 0.06 were considered significant. Several leading gene sets are displayed in the plot. **(B)** Down-regulated HALLMARK pathways in samples with low PLXDC2 expression, similar to **(A)**. **(C–F)** Kaplan–Meier plots according to the expression of DEGs within the top 10 up-regulated pathways. EMT and 3 other pathways, namely, inflammatory response, IL2-STAT5 signaling and KRAS signaling (up), were proven to be related to survival. **(G–L)** The correlation scatterplots of PLXDC2 and EMT markers. CDH2 (N-cadherin), TWIST1 and ZEB1 are up-regulated EMT markers, while CK8, CK10, and CK19 (three E- cadherin genes) are down-regulated EMT markers.

As the PLXDC2 expression level was correlated with survival time and clinicopathological characteristics, an additional GSEA was conducted to explore its relationship with HALLMARK pathways to reveal the cellular functions of PLXDC2. The samples were separates into a high expression group and a low expression group according to the median expression level of PLXDC2. 10 up-regulated pathways and 10 down-regulated pathways were then obtained. Among them, EMT was the most correlated pathway (Figures 4A,B). Survival analyses were then conducted according to the expression of DEGs in the top 10 up-regulated pathways. EMT along with three correlated immune pathways, namely, inflammatory response, IL2-STAT5 signaling and KRAS signaling (up), were identified to be survival related (Figures 4C–F), which supported the role of PLXDC2 as a gene affecting inflammatory signaling. Additional analysis was performed to study the correlation of PLXDC2 and classic EMT markers by generating correlation scatterplots, and the results were coordinate with GSEA results. The up-regulated EMT markers displayed positive correlation with PLXDC2, while its down-regulated counterpart exhibited otherwise, with a negative correlation (Figures 4G–L). These results confirmed the function of PLXDC2 in regulating EMT and inflammation.

### M2 Macrophages Gene Signatures Were Positively Associated With PLXDC2 Expression

**FIGURE 5:**
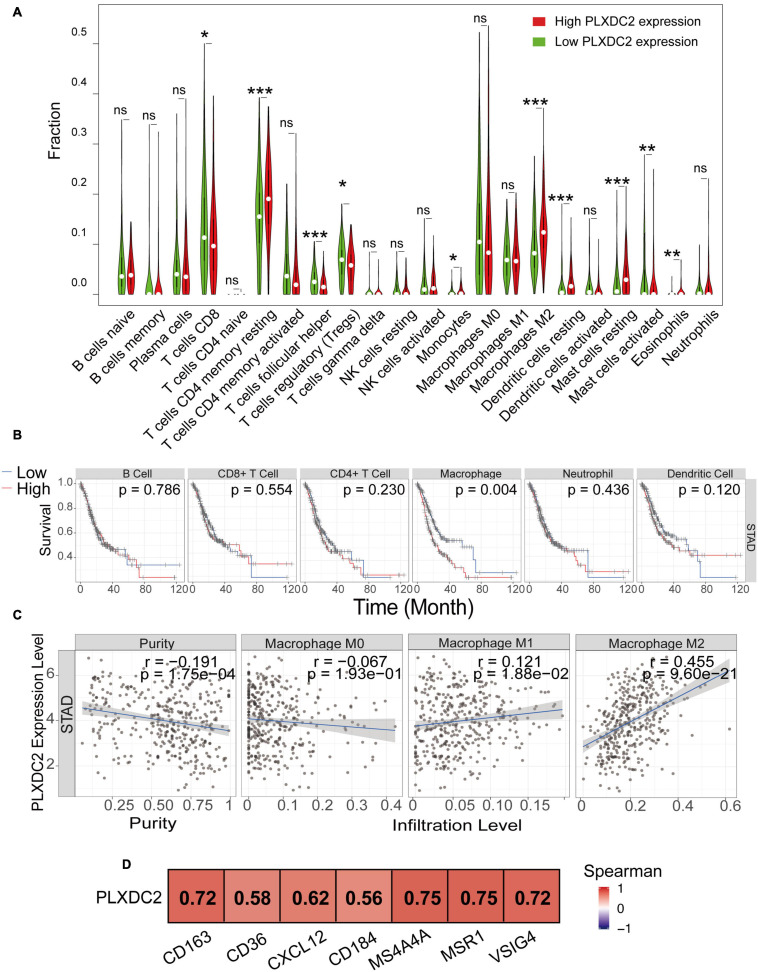
The correlation of PLXDC2 correlation with M2 macrophages and gene markers. **(A)** The ratio differentiation of 22 kinds of immune cells between TCGA STAD tumor samples with high or low PLXDC2 expression relative to the median PLXDC2 expression level; the Wilcoxon rank sum test was used as the significance test. “ns” represents *p* > 0.05, * represents 0.05 > *p* > 0.01, ** represents 0.01 > *p* > 0.001, and *** represents *p* < 0.001. **(B)** Kaplan–Meier plots according to the proportions of 6 general types of immune cells (macrophages *p* = 0.004 by the log-rank test). **(C)** Scatter plot showing the correlation of M0, M1, and M2 macrophages proportions with PLXDC2 expression (*p* < 0.05) generated by TIMER 2. The blue line in each plot was a fitted linear model that indicates the proportion of immune cells and the expression of PLXDC2; the Pearson coefficient was used for the correlation test. **(D)** The relationship between PLXDC2 and the immune markers of M2 macrophages. A redder color suggests a better correlation according to the Spearman test generated by TIMER 2.

We explored the correlation of PLXDC2 expression with the proportion of TICs subsets. The novel CIBERSORTx algorithm was applied to generate the profiles of 22 kinds of immune cells in STAD samples. STAD samples were grouped according to the median PLXDC2 expression level, and a violin plot was generated (Figure 5A). Within the 22 kinds of TICs, 5 of them were discovered as most differentially expressed (*p* < 0.001). According to the result of CIBERSORTx, the cell fractions of both T cells CD8 and Tregs are more pronounced in low PLXDC2 expression group compared to high PLXDC2 expression group, which might suggest that patients with high PLXDC2 have some suppress function to the subset T cell competent. Those results are consistent with our finding of PLXDC2 promoted CD163 M2 macrophages and our GSEA results. Those subset macrophages might trigger inflammation resolution and suppress T cell population or activation via several mechanisms (Noy and Pollard, 2014). The Tumor Immune Estimation Resource (TIMER)^8^ web tool was utilized for further exploration of the correlation between PLXDC2 and GC patients’ immune cells (Li et al., 2016, 2020). The results demonstrated that macrophages exhibited the highest correlation with PLXDC2 expression and survival rate among the cell types (Figure 5B and Supplementary Figure 2). The correlations between PLXDC2 and macrophages subtypes were also assessed (Figure 5C). Among the subtypes, M2 macrophages had the highest correlation, with a larger *R* value and a smaller *p* value. The correlation of PLXDC2 with macrophages M2 immune markers was demonstrated using the data of the “Gene-Corr” module of TIMER 2.0 (see text footnote 6; Figure 5D). These results provide solid evidence proving that the PLXDC2 expression level was closely associated with a particular immune cell type (M2 macrophages).

### Identification of PLXDC2 Upregulation in Fibroblasts and Macrophages

**FIGURE 6:**
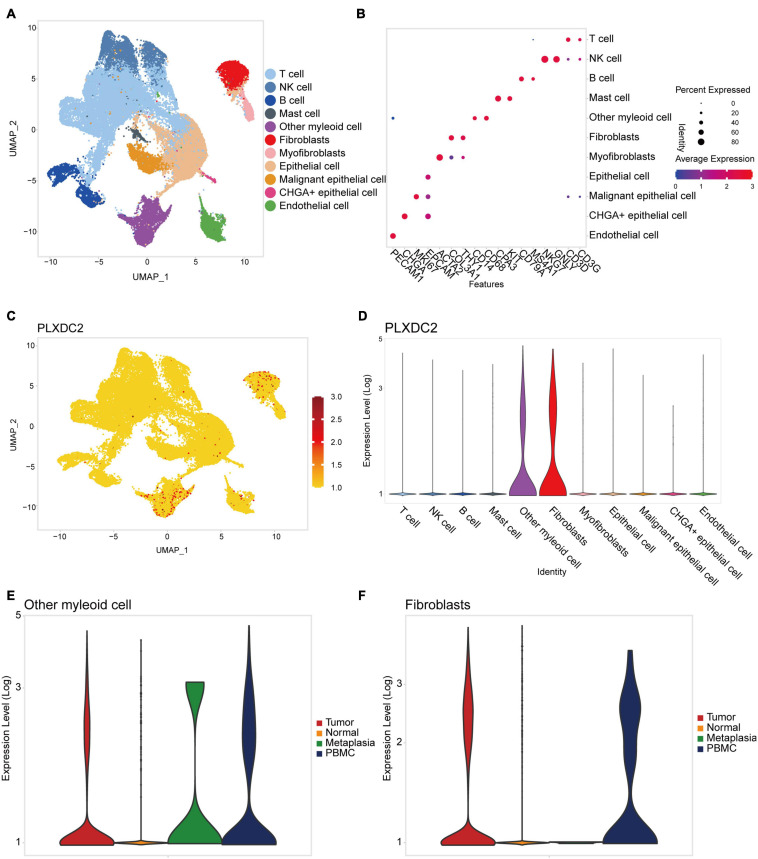
PLXDC2 expression level ascend in Fibroblasts and Macrophages. **(A)** Uniform manifold approximation and projection (UMAP) dimensionality reduction analysis. 11 cell types were identified with its unique gene marker. Other myeloid cell represents monocytes and macrophages. **(B)** Bubble plot demonstrating each cell type and its gene marker. **(C)** UMAP revealing the expression level of PLXDC2. Higher expression is indicated with a redder color. **(D)** Violin plot showing the expression of PLXDC2 in each cell type. **(E)** Violin plot showing the expression level of PLXDC2 in other myeloid cell of Metaplasia, Normal, PBMC and Tumor samples, metaplasia represents the transformation of one differentiated cell type to another differentiated cell type, and PBMC resembles peripheral blood mononuclear cell. **(F)** Violin plot showing the expression level of PLXDC2 in fibroblasts of Metaplasia, Normal, PBMC and Tumor samples, similar to **(E)**.

New efforts in single-cell profiling will enable a better understanding of the microenvironment composition in GC development. To better understand PLXDC2 expression pattern in the TME, we utilized a single-cell RNA sequence of GC. The process of the single cell analysis was demonstrated in Figures 6A,B with a Uniform Manifold Approximation and Projection (UMAP) and a bubble blot that displayed the cell markers of each cell type. The expression level of PLXDC2 in a single cell study is illustrated in Figures 6C,D. The UMAP and violin plots indicated that PLXDC2 upregulates in fibroblasts and monocytes/macrophages via unique gene markers of each cell type. Comparison of PLXDC2 expression level in different samples (metaplasia, normal, PBMC, and tumor) was established, and the result showed that PLXDC2 scarcely express in the normal sample, while upregulating in PBMC and tumor samples in both macrophages and fibroblasts, and metaplasia in macrophages (Figures 6E,F). The results revealed that PLXDC2 might be of certain significance inside a stromal environment and contribute to metaplasia in macrophages.

### Validation of the Correlations of PLXDC2 Expression and Metastatic Index and Its Prognostic Value

**FIGURE 7:**
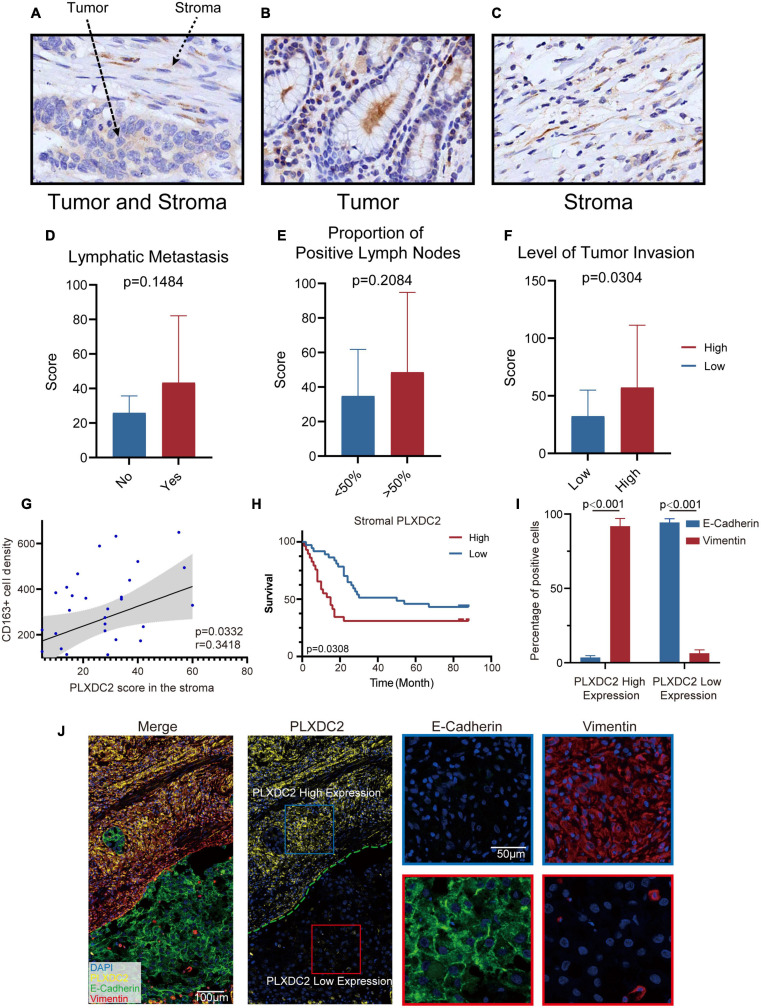
PLXDC2 upregulation in Stroma indicates poor survival and high tumor invasion level. **(A)** PLXDC2 IHC staining images of GC tumor and stromal tissues (20x). **(B)** PLXDC2 IHC staining images of GC stromal tissues (20x). **(C)** PLXDC2 IHC staining images of GC tumor tissues [20x; **(D)**] PLXDC2 staining scores of GC tumor tissues in samples group by whether lymphatic metastasis exist. Lymphatic Metastasis is consistent with N in AJCC standard. A paired *t* test was used for analysis. **(E)** PLXDC2 staining scores of GC tumor tissues in the samples group by comparing with 50% of the positive lymph nodes. The proportion of Positive Lymph Nodes refers to the proportion of positive lymph nodes in lymph nodes removed during operation. A paired *t* test was used for analysis. **(F)** PLXDC2 staining scores of GC tumor tissues in samples group by level of tumor invasion (high vs. low). Level of Tumor Invasion is consistent with T in AJCC standard. A paired *t* test was used for analysis. **(G)** Scatter plot showing the correlation of PLXDC2 score in the stroma and CD163^+^ cell density (*p* = 0.0332, *r* = 0.3418). **(H)** Kaplan–Meier plot assessing survival according to High and Low PLXDC2 score in the stroma. *p* = 0.0308 by log-rank test. **(I)** mIHC staining scores exhibiting the percentage of positive cells of GC tumor tissues group by PLXDC2 expression level [high vs. low, *p* < 0.001; **(J)**] mIHC staining images of GC section (Merge and PLXDC2-10x, E-Cadherin and Vimentin-40x).

To further study the potential clinical relevance of PLXDC2 expression in tumor progression, we made use of 67 TMA with adequate PLXDC2 and CD163 staining effect of GC patient samples to compare the expression of PLXDC2 between GC tumor tissues and stromal tissues. PLXDC2 staining was more intense in the cytoplasm than in the nucleus. The whole slide photo of the TMA and staining of CD163 is attached in Supplementary Figures 3, 4. Higher PLXDC2 expression was observed in GC stromal tissues than in tumor tissues within 52 cases, the number of which is after the removal of films with poor dyeing quality (Figures 7A–C). The comparisons of PLXDC2 expression of lymphatic metastasis (high vs. low, N1-N3b of AJCC are considered to have metastasis, N0 is considered to have no metastasis) and proportion of positive lymph node (>50% vs. <50%, within the total lymph nodes removed during operation), according to the unpaired *t* test results, showed no significant difference (Figures 7D,E). The level of tumor invasion (T4-T4b of AJCC was considered to be of level of invasion, and T0-T3 was considered to be of low level of invasion, T4 is the demarcation point between whether the tumor invades the serosa and adjacent organs) of PLXDC2 high staining score group was significantly stronger than that of PLXDC2 low staining score group (Figure 7F). The data above might lead us to the conclusion that PLXDC2 affects tumor metastasis in a non-lymphatic manner. Afterward, we studied the correlation of PLXDC2 and M2 macrophages’ gene marker of CD163. The correlation plot indicated that PLXDC2 score in stromal is linked to CD163^+^ cell density in GC, therefore correlated with M2 macrophages (Figure 7G). The information of GC patients suitable for study was demonstrated in Table 1. To test whether PLXDC2 expression could be a potential prognostic predictor in GC, a Kaplan Meier plot comparing high and low PLXDC2 score in stromal according to the medium was generated (Figure 7H). The result of the plot confirmed PLXDC2 capability to assess survival of GC patients. Immunohistochemistry method verified our *in silico* result that PLXDC2 is a vital metastatic related gene that effect within the stroma of cancer patients. The results above approved PLXDC2’s important role of promoting cancer metastasis in the stroma.

**TABLE 1:** Patients’ information.

Parameter	Stratification	Number of patients
Gender	Male	44
	Female	23
Age	<67	28
	≥67	39
Status	Deceased	42
	Survival	25
Clinical stage	1	3
	2	24
	3–4	40
PLXDC2 score	Low	17
	Medium	31
	High	19
CD163	Positive	44
	Negative	23

*Note: The information of gastric cancer patients that participated in the tissue microarray study. Gender, age, status, clinical stage, PLXDC2 score, and CD163 available.*

To further confirm the association between PLXDC2 and EMT process, mIHC was used to demonstrate the markers associated with EMT, including epithelial marker E-cadherin and mesenchymal marker Vimentin (Figures 7I,J). The staining results showed that E-cadherin was hardly expressed in the regions with high expression of PLXDC2, while Vimentin was strongly expressed. The opposite result was found in the region with low expression of PLXDC2. The mIHC results demonstrated that the overexpression of PLXDC2 was strongly correlated with the progression of EMT.

## Discussion

Gastric cancer is one of the most lethal malignancies, partly for lack of knowledge regarding the molecular patterns of GC subtypes in different stages of disease progression. Thus, this study embarked on an exploration of the TME in GC patients. Identification of PLXDC2 as a key gene was enabled with TME features of both stromal and immune domains, along with survival-related analysis. Further inquiry linked PLXDC2 with multiple clinicopathological characteristics and EMT/inflammatory signaling pathways, which implied a connection between the gene and vital clinical function. In addition, with the aid of CIBERSORTx, we discovered the linkage between PLXDC2 and a particular subtype of macrophages, M2 macrophages, which were recognized as protumor cells.

The TME comprises multiple complex components capable of suppressing and promoting tumor growth and metastasis. The TME has been identified to contribute to cell growth in several tumors, as it protects tumor cells from the attack of tumor-infiltrating lymphocytes (TILs; Peranzoni et al., 2018). In our study, we assessed TME characteristics via ESTIMATE and CIBERSORTx. The former computed the ratio of immune and stromal components, which was utilized to screen and identify key genes. The latter calculated the TICs proportions, which contributed to exploring key genes linked with M2 macrophages. More recent study reported that scRNA-seq highlights the role of inflammatory cancer-associated fibroblasts in bladder urothelial carcinoma (Chen et al., 2020). TME study based on single-cell RNA sequence also aided us in exploring GC TME. Based on the given estimations, we discovered that a high stromal score could be a potential indicator of a poor prognosis in GC patients.

Furthermore, with the novel CIBERSORTx tool, we identified the close correlation between our key gene (PLXDC2) and a particular subtype of immune cells (M2 macrophages). Consistent with previous studies, a high proportion of M2 macrophages in the TME of GC was an unfavorable prognostic factor, which matched the influence of PLXDC2 on survival. Our study demonstrated the linkage of PLXDC2 and M2 macrophages and may encourage PLXDC2 and M2 macrophages markers’ clinical application in prognosis evaluation.

Plexin domain containing 2 is a vital member of the TEM family and is associated with tumor-specific angiogenesis. It encodes a type I transmembrane protein with some homology to nidogen and to plexins. Previous studies implied that it is an additional component in the network of proteins regulating proliferation and differentiation in the developing nervous system. While the functions of PLXDC2 in some tumors have been explored and demonstrated to have potential significance in the progression and molecular targeting of cancers such as colorectal cancer, few studies have focused on the behavior of PLXDC2 in GC, and our study addressed this gap (Rmali et al., 2005). Our TME-based study revealed the potential prognostic value of PLXDC2 in GC stroma as an indicator of poor outcomes, which may provide a novel line of thinking around GC prognosis evaluation. Despite the fact that PLXDC2 demonstrated a correlation with several clinicopathological characteristics and was revealed as a key regulator that promotes GC progression, the gene was discovered to have a low correlation with previously identified immune checkpoints, such as PD-L1, which may indicate that PLXDC2 acts independently of well-known immune regulatory genes, such as PD-L1. Previous studies concluded that deficiency of PLXDC2 is associated with increased inflammation, but our study shows otherwise. GSEA results in our study indicated that elevation of PLXDC2 in a GC cohort was linked with increased inflammation. Our results challenge the previous belief and may enable us to comprehend the role of PLXDC2 in inflammatory signaling while provoking further studies on this topic. The function of PLXDC2 in promoting angiogenesis has recently been discovered. Our study confirmed this previous finding and identified a new EMT-related function for PLXDC2. Epithelial-mesenchymal transition in GC have been discussed in previous studies of our lab, and this study extended our understanding of EMT in GC (Liu et al., 2021). The function of our key gene in EMT may indicate that PLXDC2 is a regulator of tumor metastasis. It has also been reported that deficiency of PLXDC2 suppresses the bacterial colonization of HP infection (Tubau-Juni et al., 2020). In our studies, however, we discovered no clear evidence that the expression level of PLXDC2 may influence HP infection in GC (Supplementary Figure 2). The relationship between PLXDC2 and HP infection could be a topic requiring further exploration. In contrast to a recent study, despite being the closest homolog of PLXDC2, PLXDC1 was not significantly differentially expressed between tumor samples and normal samples at the gene level, leaving PLXDC2 as the only TEM suitable for predicting the survival outcomes of GC (Zhang et al., 2015).

As a major component of innate immune cells, macrophages exert great influence in the TME (Davies et al., 2013). The accumulation of certain types of macrophages can indicate poor outcomes, as they hinder CD8^+^ T lymphocytes’ function. M2 macrophages, or alternatively activated macrophages, have been confirmed by multiple studies to play a decisive role in promoting tumor growth and cancer development. Tumor associated macrophages’ behavior pattern is a topic that our group previously looked into (Wu et al., 2020). As a portion of our study, we confirmed the protumor function of M2 macrophages and linked this particular type of macrophages with the expression of our key gene PLXDC2. The gene exhibited a strong correlation with M2 macrophages and their markers, such as CD163. In addition to the fact that PLXDC2 gene expression shows a strong correlation with cytokines’ expression levels, such as IL10, the linkage of PLXDC2 and macrophages demonstrated in this study might suggest that PLXDC2 plays some role in recruiting M2 macrophages. Single-cell analysis of our study confirmed PLXDC2’s upregulation in Macrophages. On the other hand, the phenotype of M2 macrophages was not enough to explain the function of our key gene PLXDC2 in GC samples. M2 macrophages have been well recognized as anti-inflammatory components, whereas our gene PLXDC2 demonstrated proinflammatory behaviors according to GSEA (Orecchioni et al., 2019). This might bring us to the conclusion that our gene does not influence the phenotype of M2 macrophages in an inflammatory form. Further exploration of the inflammatory function of PLXDC2 will be needed for a rigorous conclusion. Both our TCGA study and IHC study found significance in comparison of high and low levels of tumor invasion but not in metastasis through lymph nodes. Along with single-cell study’s result that PLXDC2 is an important stromal related cell and the GSEA result that PLXDC2 affects EMT, our results point to the fact that PLXDC2 supports tumor invasion via a non-lymphatic, EMT related method.

## Conclusion

Our study identified the TME-related gene PLXDC2 in a high-stromal population and had a prognostic signature correlated to the CD163 M2 macrophages, further promoting EMT markers. Similar to the previous reporting that stromal EMT gene signature affects GC prognosis and immunotherapy responsiveness (Zheng and Li, 2020; Liu et al., 2021). The combined PLXDC2/EMT/M2 macrophages axis may affect GC outcome and immunotherapy responsiveness. The crosstalk between PLXDC2 expressed tumor stroma and CD163 macrophages and with tumor cell’s EMT process is extricate and interesting. Further study of stromal PLXDC2, CD163 related EMT, and their targeted genes will help provide potential novel targets for GC.

## Data Availability Statement

The datasets presented in this study can be found in online repositories. The names of the repository/repositories and accession number(s) can be found in the article/Supplementary Material.

## Ethics Statement

The studies involving human participants were reviewed and approved by Ethics Committee of Shanghai Jiao Tong University. The patients/participants provided their written informed consent to participate in this study.

## Author Contributions

YH and DX contributed to the conception and design of the research work. YG, YD, GW, HG, YX, EL, and JX performed the experiments and performed data acquisition and analysis and interpretation. YG, YH, and DX drafted the article. PX, PN, DW, DX, and YH provided critical reagents and supervised the research. All authors have read and agreed to the published version of the manuscript.

## Conflict of Interest

The authors declare that the research was conducted in the absence of any commercial or financial relationships that could be construed as a potential conflict of interest.

## Footnotes

1 https://dna-discovery.stanford.edu/research/datasets/

2 https://bioinformatics.mdanderson.org/estimate/

3 http://gepia2.cancer-pku.cn/#index

4 http://www.kmplot.com/analysis/

5 https://cibersortx.stanford.edu

6 http://timer.cistrome.org

7 https://www.10xgenomics.com/products/single-cell-gene-expression

8 https://cistrome.shinyapps.io/timer/

## References

[B1] Bray F. Ferlay J. Soerjomataram I. Siegel R. L. Torre L. A. Jemal A. (2018). Global cancer statistics 2018: GLOBOCAN estimates of incidence and mortality worldwide for 36 cancers in 185 countries. *CA Cancer J. Clin.* 68 394–424. 10.3322/caac.21492 30207593

[B2] Chen W. Zheng R. Baade P. D. Zhang S. Zeng H. Bray F. (2016). Cancer statistics in China, 2015. *CA Cancer J. Clin.* 66 115–132. 10.3322/caac.21338 26808342

[B3] Chen Y. C. Suresh A. Underbayev C. Sun C. Singh K. Seifuddin F. (2019). IKAP-Identifying K mAjor cell population groups in single-cell RNA-sequencing analysis. *Gigascience* 8:giz121. 10.1093/gigascience/giz121 PMC6771546 31574155

[B4] Chen Z. Zhou L. Liu L. Hou Y. Xiong M. Yang Y. (2020). Single-cell RNA sequencing highlights the role of inflammatory cancer-associated fibroblasts in bladder urothelial carcinoma. *Nat. Commun.* 11: 5077. 10.1038/s41467-020-18916-5 PMC7545162 33033240

[B5] Davies L. C. Jenkins S. J. Allen J. E. Taylor P. R. (2013). Tissue-resident macrophages. *Nat. Immunol.* 14 986–995. 24048120 10.1038/ni.2705 PMC4045180

[B6] Edge S. B. Compton C. C. (2010). The American joint committee on cancer: the 7th edition of the AJCC cancer staging manual and the future of TNM. *Ann. Surg. Oncol.* 17 1471–1474. 20180029 10.1245/s10434-010-0985-4

[B7] Hatakeyama M. (2019). Malignant *Helicobacter pylori*-associated diseases: gastric cancer and MALT Lymphoma. *Adv. Exp. Med. Biol.* 1149 135–149. 10.1007/5584_2019_363 31016622

[B8] Huber W. Carey V. J. Gentleman R. Anders S. Carlson M. Carvalho B. S. (2015). Orchestrating high-throughput genomic analysis with Bioconductor. *Nat. Methods* 12 115–121. 10.1038/nmeth.3252 25633503 PMC4509590

[B9] Jackaman C. Tomay F. Duong L. Abdol Razak N. B. Pixley F. J. Metharom P. (2017). Aging and cancer: the role of macrophages and neutrophils. *Age. Res. Rev.* 36 105–116. 10.1016/j.arr.2017.03.008 28390891

[B10] Kotilea K. Bontems P. Touati E. (2019). Epidemiology, diagnosis and risk factors of *Helicobacter pylori* infection. *Adv. Exp. Med. Biol.* 1149 17–33. 10.1007/5584_2019_357 31016621

[B11] Krebs A. M. Mitschke J. Lasierra Losada M. Schmalhofer O. Boerries M. Busch H. (2017). The EMT-activator Zeb1 is a key factor for cell plasticity and promotes metastasis in pancreatic cancer. *Nat. Cell Biol.* 19 518–529. 10.1038/ncb3513 28414315

[B12] Lewis C. E. Pollard J. W. (2006). Distinct role of macrophages in different tumor microenvironments. *Cancer Res.* 66 605–612. 10.1158/0008-5472.can-05-4005 16423985

[B13] Li B. Severson E. Pignon J. C. Zhao H. Li T. Novak J. (2016). Comprehensive analyses of tumor immunity: implications for cancer immunotherapy. *Genome Biol.* 17:174. 10.1186/s13059-016-1028-7 PMC4993001 27549193

[B14] Li T. Fu J. Zeng Z. Cohen D. Li J. Chen Q. (2020). TIMER2.0 for analysis of tumor-infiltrating immune cells. *Nucleic Acids Res.* 48 W509–W514. 32442275 10.1093/nar/gkaa407 PMC7319575

[B15] Liu C. Deng L. Lin J. Zhang J. Huang S. Zhao J. (2021). Zinc finger protein CTCF Regulates Extracellular Matrix (ECM)-related gene expression associated with the Wnt signaling pathway in gastric cancer. *Front. Oncol.* 10:625633. 10.3389/fonc.2020.625633 33665169 PMC7921701

[B16] McGinnis C. S. Murrow L. M. Gartner Z. J. (2019). DoubletFinder: doublet detection in single-cell RNA sequencing data using artificial nearest neighbors. *Cell Syst.* 8 329–337.e4. 30954475 10.1016/j.cels.2019.03.003 PMC6853612

[B17] Miller-Delaney S. F. Lieberam I. Murphy P. Mitchell K. J. (2011). Plxdc2 is a mitogen for neural progenitors. *PLoS One* 6:e14565. 10.1371/journal.pone.0014565 21283688 PMC3024984

[B18] Nanda A. Buckhaults P. Seaman S. Agrawal N. Boutin P. Shankara S. (2004). Identification of a binding partner for the endothelial cell surface proteins TEM7 and TEM7R. *Cancer Res.* 64 8507–8511. 10.1158/0008-5472.can-04-2716 15574754

[B19] Noy R. Pollard J. W. (2014). Tumor-associated macrophages: from mechanisms to therapy. *Immunity* 41 49–61. 10.1016/j.immuni.2014.06.010 25035953 PMC4137410

[B20] Orecchioni M. Ghosheh Y. Pramod A. B. Ley K. (2019). Macrophage polarization: different gene signatures in M1(LPS+) vs. Classically and M2(LPS-) vs. alternatively activated macrophages. *Front. Immunol.* 10:1084. 10.3389/fimmu.2019.01084 31178859 PMC6543837

[B21] Pastushenko I. Blanpain C. E. M. T. (2019). Transition states during tumor progression and metastasis. *Trends Cell Biol.* 29 212–226. 10.1016/j.tcb.2018.12.001 30594349

[B22] Peranzoni E. Lemoine J. Vimeux L. Feuillet V. Barrin S. Kantari-Mimoun C. (2018). Macrophages impede CD8 T cells from reaching tumor cells and limit the efficacy of anti-PD-1 treatment. *Proc. Natl. Acad. Sci. U.S.A.* 115 E4041–E4050. 29632196 10.1073/pnas.1720948115 PMC5924916

[B23] Ritchie M. E. Phipson B. Wu D. Hu Y. Law C. W. Shi W. (2015). limma powers differential expression analyses for RNA-sequencing and microarray studies. *Nucleic Acids Res.* 43:e47. 10.1093/nar/gkv007 25605792 PMC4402510

[B24] Rmali K. A. Puntis M. C. Jiang W. G. (2005). Prognostic values of tumor endothelial markers in patients with colorectal cancer. *World J. Gastroenterol.* 11 1283–1286. 10.3748/wjg.v11.i9.1283 15761964 PMC4250673

[B25] Sathe A. Grimes S. M. Lau B. T. Chen J. Suarez C. Huang R. J. (2020). Single-Cell genomic characterization reveals the cellular reprogramming of the gastric tumor microenvironment. *Clin. Cancer Res.* 26 2640–2653. 10.1158/1078-0432.ccr-19-3231 32060101 PMC7269843

[B26] Siegel R. L. Miller K. D. Fuchs H. E. Jemal A. (2021). Cancer statistics, 2021. *CA Cancer J. Clin.* 71 7–33. 33433946 10.3322/caac.21654

[B27] Steen C. B. Liu C. L. Alizadeh A. A. Newman A. M. (2020). Profiling cell type abundance and expression in bulk tissues with CIBERSORTx. *Methods Mol. Biol.* 2117 135–157. 10.1007/978-1-0716-0301-7_7 31960376 PMC7695353

[B28] Stuart T. Butler A. Hoffman P. Hafemeister C. Papalexi E. Mauck W. M. III (2019). Comprehensive integration of single-cell data. *Cell* 177 1888–1902.e21. 31178118 10.1016/j.cell.2019.05.031 PMC6687398

[B29] Subramanian A. Tamayo P. Mootha V. K. Mukherjee S. Ebert B. L. Gillette M. A. (2005). Gene set enrichment analysis: a knowledge-based approach for interpreting genome-wide expression profiles. *Proc. Natl. Acad. Sci. U.S.A.* 102 15545–15550. 10.1073/pnas.0506580102 16199517 PMC1239896

[B30] Szasz A. M. Lanczky A. Nagy A. Forster S. Hark K. Green J. E. (2016). Cross-validation of survival associated biomarkers in gastric cancer using transcriptomic data of 1,065 patients. *Oncotarget* 7 49322–49333. 10.18632/oncotarget.10337 27384994 PMC5226511

[B31] Szebeni G. J. Vizler C. Kitajka K. Puskas L. G. (2017). Inflammation and cancer: extra- and intracellular determinants of tumor-associated macrophages as tumor promoters. *Med. Inflamm.* 2017:9294018. 10.1155/2017/9294018 PMC5286482 28197019

[B32] Tang Z. Kang B. Li C. Chen T. Zhang Z. (2019). GEPIA2: an enhanced web server for large-scale expression profiling and interactive analysis. *Nucleic Acids Res.* 47 W556–W560. 31114875 10.1093/nar/gkz430 PMC6602440

[B33] Tubau-Juni N. Bassaganya-Riera J. Leber A. Zoccoli-Rodriguez V. Kronsteiner B. Viladomiu M. (2020). Identification of new regulatory genes through expression pattern analysis of a global RNA-seq dataset from a *Helicobacter pylori* co-culture system. *Sci. Rep.* 10:11506. 10.1038/s41598-020-68439-8 PMC7359330 32661418

[B34] Wu K. Lin K. Li X. Yuan X. Xu P. Ni P. (2020). Redefining tumor-associated macrophage subpopulations and functions in the tumor microenvironment. *Front. Immunol.* 11:1731. 10.3389/fimmu.2020.01731 32849616 PMC7417513

[B35] Wu T. Dai Y. (2017). Tumor microenvironment and therapeutic response. *Cancer Lett.* 387 61–68. 10.1016/j.canlet.2016.01.043 26845449

[B36] Yamamoto N. Eguchi A. Hirokawa Y. Ogura S. Sugimoto K. Iwasa M. (2020). Expression pattern of plexin domain containing 2 in human hepatocellular carcinoma. *Monoclon. Antib. Immunodiagn. Immunother.* 39 57–60. 10.1089/mab.2019.0050 32202949

[B37] Yasui W. Oue N. Kuniyasu H. Ito R. Tahara E. Yokozaki H. (2001). Molecular diagnosis of gastric cancer: present and future. *Gastr. Cancer* 4 113–121. 10.1007/pl00011733 11760076

[B38] Yoon S. J. Park J. Shin Y. Choi Y. Park S. W. Kang S. G. (2020). Deconvolution of diffuse gastric cancer and the suppression of CD34 on the BALB/c nude mice model. *BMC Cancer* 20:314. 10.1186/s12885-020-06814-4 32293340 PMC7160933

[B39] Yoshihara K. Shahmoradgoli M. Martinez E. Vegesna R. Kim H. Torres-Garcia W. (2013). Inferring tumour purity and stromal and immune cell admixture from expression data. *Nat. Commun.* 4:2612. 10.1038/ncomms3612 PMC3826632 24113773

[B40] Yunna C. Mengru H. Lei W. Weidong C. (2020). Macrophage M1/M2 polarization. *Eur. J. Pharmacol.* 877:173090. 10.1016/j.ejphar.2020.173090 32234529

[B41] Zeng D. Li M. Zhou R. Zhang J. Sun H. Shi M. (2019). Tumor microenvironment characterization in gastric cancer identifies prognostic and immunotherapeutically relevant gene signatures. *Cancer Immunol. Res.* 7 737–750. 10.1158/2326-6066.cir-18-0436 30842092

[B42] Zhang Y. Weinberg R. A. (2018). Epithelial-to-mesenchymal transition in cancer: complexity and opportunities. *Front. Med.* 12 361–373. 10.1007/s11684-018-0656-6 30043221 PMC6186394

[B43] Zhang Z. Z. Hua R. Zhang J. F. Zhao W. Y. Zhao E. H. Tu L. (2015). TEM7 (PLXDC1), a key prognostic predictor for resectable gastric cancer, promotes cancer cell migration and invasion. *Am. J. Cancer Res.* 5 772–781. 25973314 PMC4396023

[B44] Zheng P. Li W. (2020). Crosstalk between mesenchymal stromal cells and tumor-associated macrophages in gastric cancer. *Front. Oncol.* 10:571516. 10.3389/fonc.2020.571516 33163402 PMC7581781

[B45] Zong L. Abe M. Seto Y. Ji J. (2016). The challenge of screening for early gastric cancer in China. *Lancet* 388:2606. 10.1016/s0140-6736(16)32226-7 27894662

