# Supplementary material for: Overexpression of PLXDC2 in Stromal Cell-Associated M2 Macrophages Is Related to EMT and the Progression of Gastric Cancer
Source: Front Cell Dev Biol. 2021 May 28;9:673295. doi: 10.3389/fcell.2021.673295 (PMC8194078; doi:10.3389/fcell.2021.673295)
Supplement: Supplementary file 1 [file Data_Sheet_1.pdf]

Overexpression of PLXDC2 in Stromal Cell-Associated M2 Macrophages is Related to EMT and the Progression of Gastric Cancer

SUPPLEMENTARY MATERIALS

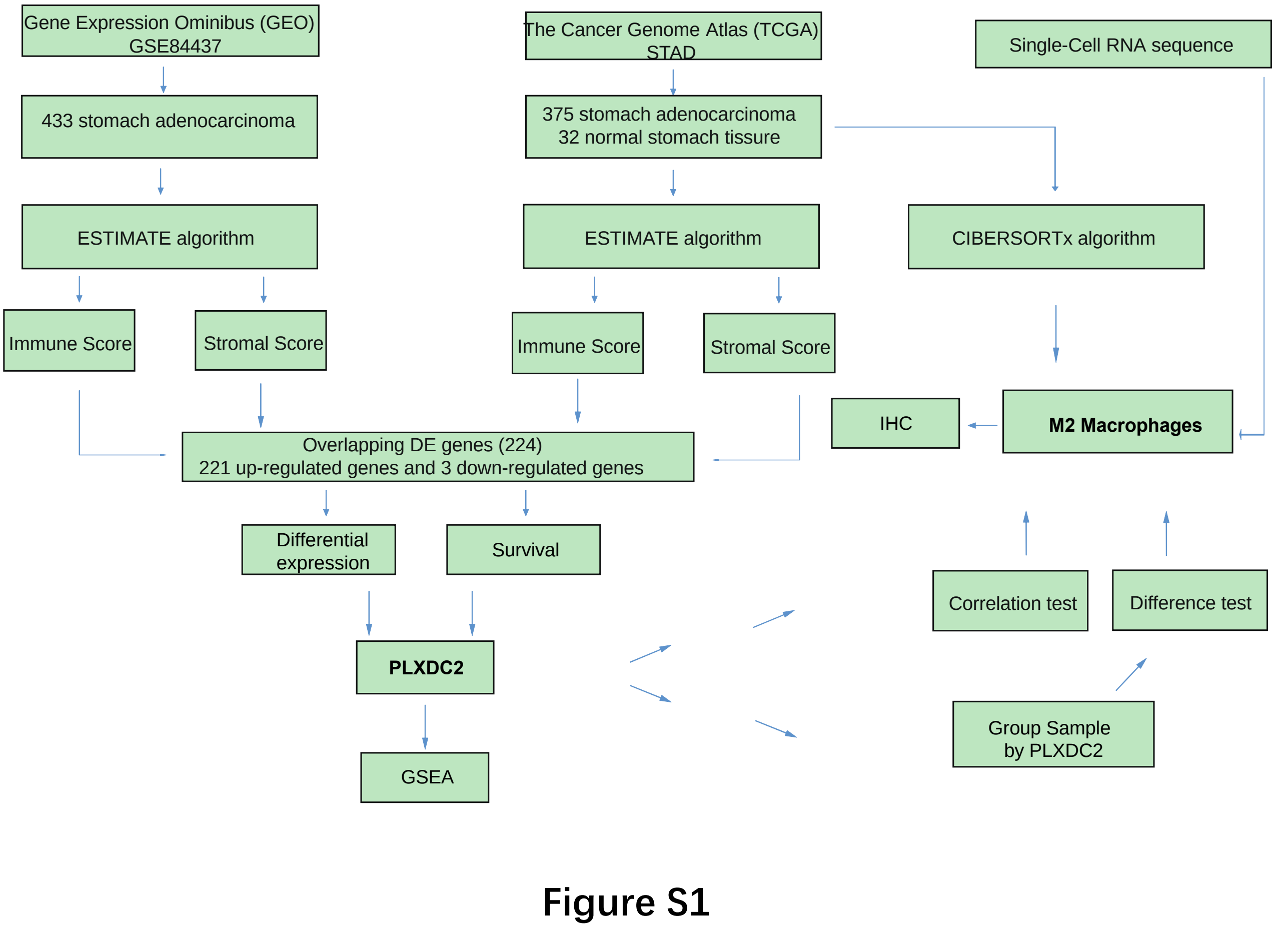

Figure S1: Workflow of this study.

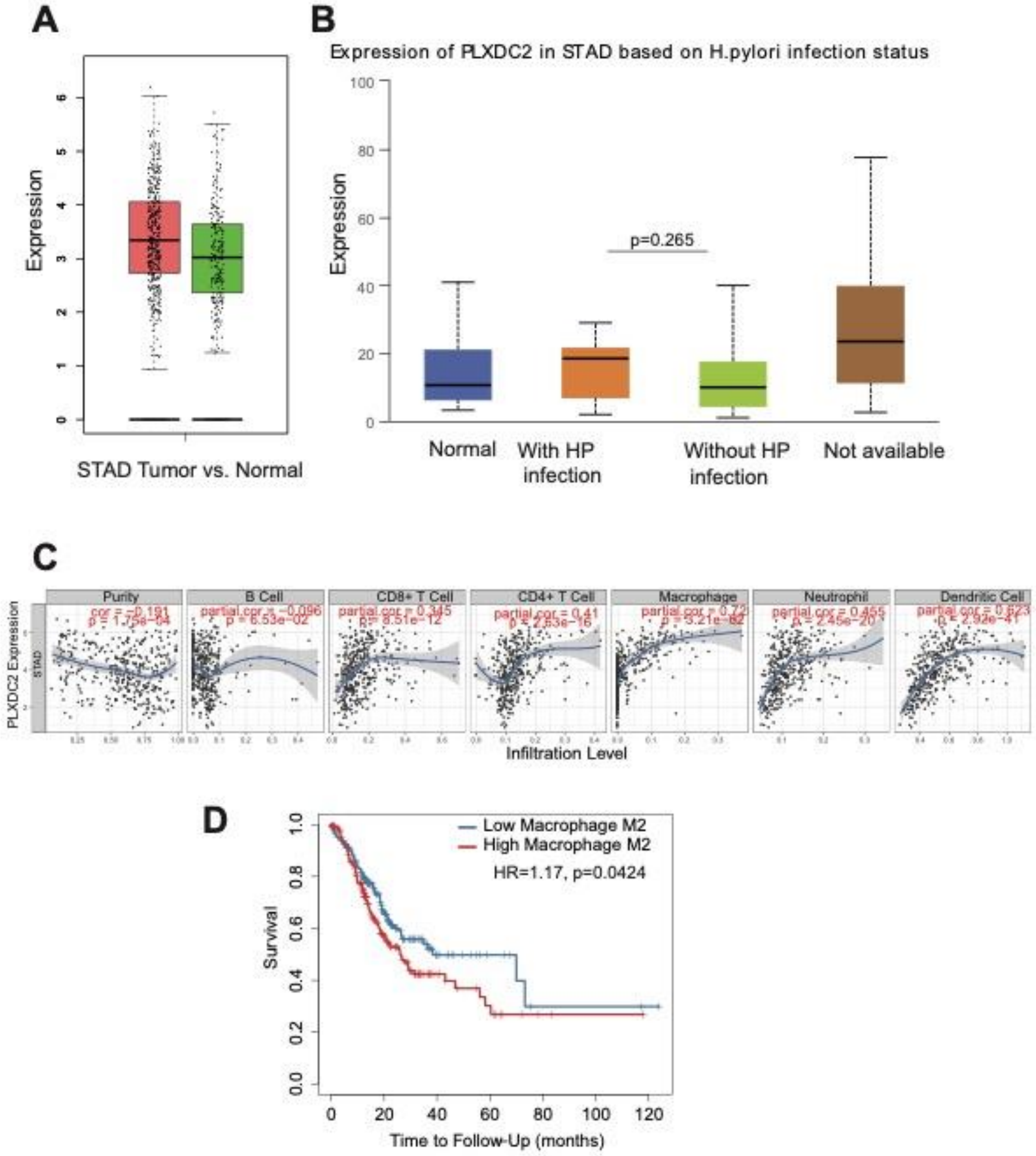

Figure S2

Figure S2: (A) Comparison of PLXDC1 expression between tumor and normal samples. (B) Comparison of PLXDC2 expression between samples with and without HP. Infection. (C) Correlation scatter plot of PLXDC2 and 6 general immune cell types. (D) Survival KM plot of M2 Macrophage, p=0.0424 by log-rank test.

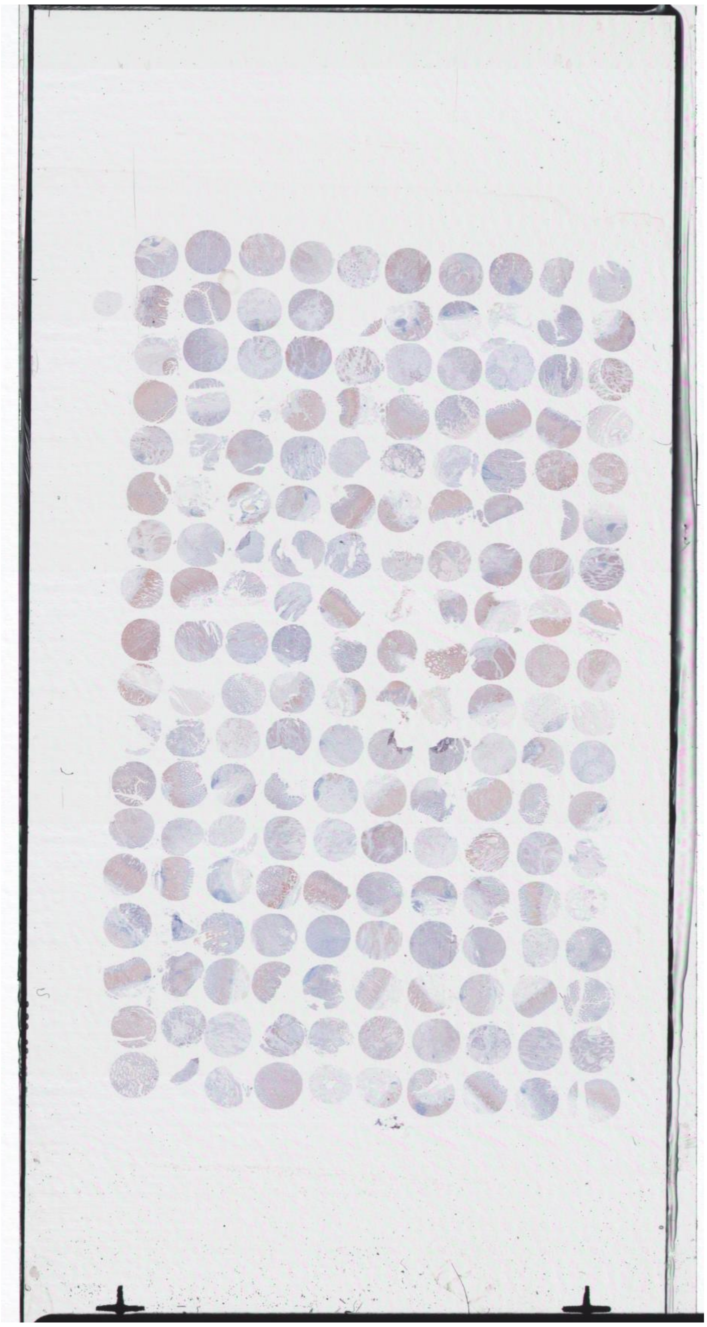

Figure S3

Figure S3: The whole slide photo of the TMA.

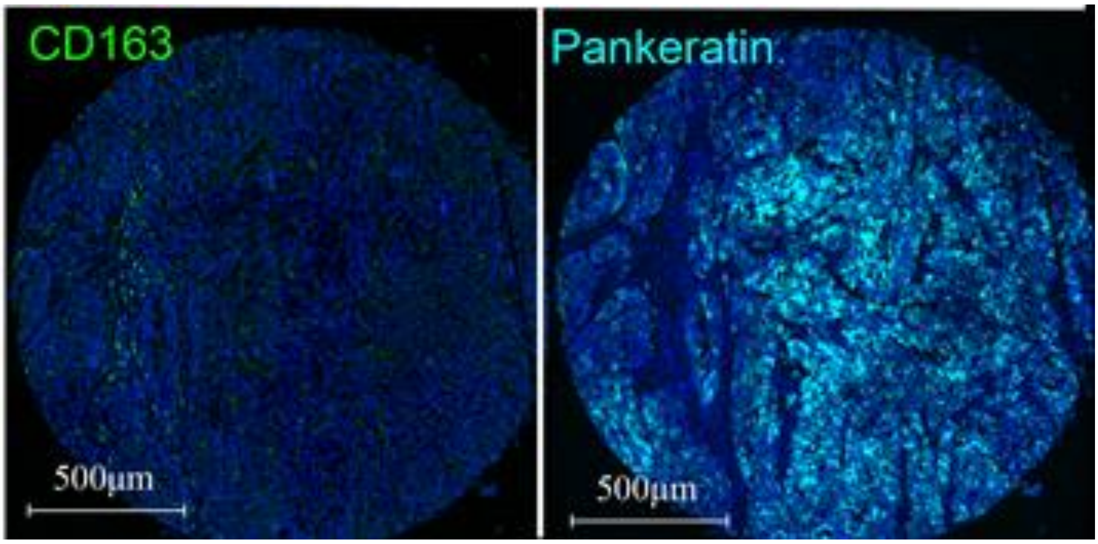

Figure S4

Figure S4: The slide photo of the TMA, displaying CD163 and Pankeratin staining.
